# On ERPs detection in disorders of consciousness rehabilitation

**DOI:** 10.3389/fnhum.2013.00775

**Published:** 2013-11-20

**Authors:** Monica Risetti, Rita Formisano, Jlenia Toppi, Lucia R. Quitadamo, Luigi Bianchi, Laura Astolfi, Febo Cincotti, Donatella Mattia

**Affiliations:** ^1^Neuroelectrical Imaging and BCI Laboratory, Fondazione Santa LuciaRome, Italy; ^2^Post-Coma Unit, Fondazione Santa LuciaRome, Italy; ^3^Department of Computer, Control, and Management Engineering, Sapienza UniversityRome, Italy; ^4^Department of Electronic Engineering, “Tor Vergata” UniversityRome, Italy; ^5^Department of Civil Engineering and Computer Science Engineering, “Tor Vergata” UniversityRome, Italy

**Keywords:** acquired brain injury, consciousness, vegetative state, minimally conscious state, ERP, P300, CRS-R

## Abstract

Disorders of Consciousness (DOC) like Vegetative State (VS), and Minimally Conscious State (MCS) are clinical conditions characterized by the absence or intermittent behavioral responsiveness. A neurophysiological monitoring of parameters like Event-Related Potentials (ERPs) could be a first step to follow-up the clinical evolution of these patients during their rehabilitation phase. Eleven patients diagnosed as VS (*n* = 8) and MCS (*n* = 3) by means of the JFK Coma Recovery Scale Revised (CRS-R) underwent scalp EEG recordings during the delivery of a 3-stimuli auditory *oddball* paradigm, which included standard, deviant tones and the subject own name (SON) presented as a *novel* stimulus, administered under passive and active conditions. Four patients who showed a change in their clinical status as detected by means of the CRS-R (i.e., moved from VS to MCS), were subjected to a second EEG recording session. All patients, but one (anoxic etiology), showed ERP components such as mismatch negativity (MMN) and novelty P300 (nP3) under passive condition. When patients were asked to count the *novel* stimuli (active condition), the nP3 component displayed a significant increase in amplitude (*p* = 0.009) and a wider topographical distribution with respect to the passive listening, only in MCS. In 2 out of the 4 patients who underwent a second recording session consistently with their transition from VS to MCS, the nP3 component elicited by passive listening of SON stimuli revealed a significant amplitude increment (*p* < 0.05). Most relevant, the amplitude of the nP3 component in the active condition, acquired in each patient and in all recording sessions, displayed a significant positive correlation with the total scores (*p* = 0.004) and with the auditory sub-scores (*p* < 0.00001) of the CRS-R administered before each EEG recording. As such, the present findings corroborate the value of ERPs monitoring in DOC patients to investigate residual *unconscious and conscious* cognitive function.

## Introduction

Disorders of Consciousness (DOC), such as Vegetative State (VS), and Minimally Conscious State (MCS) can be the consequence of severe acquired brain injury (such as traumatic brain injury, cerebral anoxia, stroke, toxic brain lesions, and encephalitis) and they usually follow a period of coma (Bernat, 2006; Goldfine and Schiff, 2011). During clinical assessment, patients with DOC typically display the absence or the inconsistency of overt behavioral responses to external stimulation. This unresponsiveness leads to consider them as lacking awareness of themselves and their environment. This approach based on negative evidence might, however, lead to a not negligible rate of diagnostic errors (Andrews et al., 1996; Schnakers et al., 2009a; Cruse et al., 2011).

Recently, the application of neuroimaging techniques such as functional magnetic resonance imaging to DOC patients with a lack of- or minimal responsiveness has provided a promising means for detecting “residual” conscious awareness otherwise not revealed by means of standard clinical approaches (Owen et al., 2006; Owen and Coleman, 2008; Monti et al., 2010; Laureys and Schiff, 2012). Alongside behavioral assessment and functional neuroimaging approaches, the electroencephalographic (EEG) technique has been shown to increase the probability to unveil possible residual covert awareness in each of these patients, with the advantage of an extreme versatile and affordable technique (Babiloni et al., 2009; Cruse et al., 2011; Goldfine et al., 2011; Cruse et al., 2012). To what extent the EEG activity changes in response to several paradigms applied to DOC patients (especially those in VS) allow to unambiguously establish covert awareness, still remains a matter of debate (Goldfine et al., 2012).

In the wide spectrum of the EEG brain signals, event-related potentials (ERPs) are recognized as a cornerstone to assess information processing ability in the absence of explicit behavior (Donchin et al., 1978; Vanhaudenhuyse et al., 2008; Lehembre et al., 2012). Despite the clinical and prognostic significance of ERP presence being currently under definition, the elicitation of such EEG brain responses is an effective sign to immediately identify patients who are responsive on a cortical level. In particular, the presence of long-latency ERP components, involving fronto-temporo-parietal cortices and backward connection between these areas, has been described as a reliable marker for neuronal conscious perception (Del Cul et al., 2007; Garrido et al., 2007; Boly et al., 2011). The elicitation of ERP after brain injury can also help predict the subsequent recovery of consciousness and, if systematically assessed, can suggest new rehabilitation strategies (Steppacher et al., 2013).

In the light of these considerations, the aims of this study were to monitor auditory ERP's components detected in a group of DOC patients during their rehabilitative treatment, and ultimately to correlate ERP components with the outcome of the clinical assessment performed by means of the gold-standard JFK Coma Recovery Scale Revised (CRS-R) (Kalmar and Giacino, 2005; Lombardi et al., 2007). The relevance of this longitudinal pilot study resides in fostering the introduction of a quantifiable measure to assess patients' motor-independent responses to commands during their rehabilitation treatment.

We administered an auditory paradigm to elicit ERP components such as P300 (Sutton et al., 1965; Squires et al., 1975; Friedman et al., 2001) and the mismatch negativity (MMN) (Näätänen et al., 1978; Tiitinen et al., 1994; Boly et al., 2011). These cognitive ERP components offer the opportunity to explore the patient's automatic attentional (pre-attentive) and attentive resources through the easiest accessible sensory modality. To this purpose, we chose a 3-stimuli *oddball* paradigm composed by standard, deviant tones and also the subject own name (SON), this latter being introduced as a novel stimulus (Holeckova et al., 2006; Perrin et al., 2006; Fischer et al., 2008, 2010; Qin et al., 2008). Such SON stimulus seems to amplify the response normally elicited by novel simple tones (less frequent than deviants), likely being characterized by a greater acoustic complexity (Kotchoubey et al., 2004) associated with a strong semantic/ecological salience (Perrin et al., 2006). Although it should be considered that the response elicited by SON stimuli in DOC patients is not name-specific, there is repeated evidence of a robust P300 when the subjects were asked to count the own name and thus, it still remains a valuable instrument to probe possible signs of residual “command-following” in these patients (Schnakers et al., 2008, 2009b; Lehembre et al., 2012).

Understanding to what extent changes in clinical indices (CRS-R subscales scores) observed during rehabilitation may have a counter part in the neurophysiological parameters would facilitate the elaboration of an integrated protocol for the assessment of patients with DOC. In addition, monitoring such electrophysiological brain responses represents the initial step to unmask relevant EEG activity required to use an EEG-based Brain Computer Interface (BCI) as a tool for a basic form of communication (Wolpaw et al., 2002).

## Materials and methods

### Patients

Sixteen patients were consecutively recruited at the Post-Coma Unit of the Neurorehabilitation Hospital “Fondazione Santa Lucia” (Rome) at the time of admission. We excluded one patient with bilaterally absent Brainstem Auditory Evoked Responses (BAERs, see paragraph EEG data acquisition), and four patients from whom we could not acquire a usable EEG-signal, due to decompressive craniectomy, vegetative dysauthonomia (profuse sweating) and psychomotor agitation. All 11 patients enrolled in the study had a history of severe acquired brain injury (Glasgow Coma Scale = 8 in the acute phase; Teasdale and Jennett, 1974; Jennett et al., 1976) and met the CRS-R diagnosis of VS and MCS. The CRS-R is a standardized and validated behavioral assessment scale to determine the patients' level of consciousness. It assesses auditory, visual, verbal and motor function as well as communication and arousal level, with a total score ranging between 0 (coma) and 23 (emergence from MCS). Patients' demographic, clinical and CRS-R sub-scores are reported in Table 1. Four out of 7 patients (patients 2, 7, 8, 11 in Table 1) underwent a second EEG recording session at the time of a detectable evolution from VS to MCS, according to the daily clinical assessment (i.e., CRS-R).

**Table 1:** **Demographic and clinical data of the study sample of Vegetative State (VS) and Minimally Conscious State (MCS) patients**.

**Patient**	**Gender**	**Age**	**Etiology**	**Months from event**	**CRS-r sub-scores**	**Diagnosis**
1	Male	30	Traumatic Brain injury	13	3–3–2–0–0–2	MCS
2	Female	21	Traumatic	12	2–1–2–2–0–1	VS
			Brain injury	16	4–2–3–1–0–2^*^	MCS
4	Male	43	Hemorrhagic stroke	19,5	3–4–4–2–1–2	MCS
5	Female	45	Hemorrhagic stroke	15	1–1–2–0–0–2	VS
6	Male	25	Hemorrhagic stroke	5	1–1–2–1–0–2	VS
7	Female	50	Cerebral	5	1–1–2–2–0–2	VS
			Anoxia	8	3–1–4–3–1–2^*^	MCS
8	Male	22	Traumatic	4	2–1–1–1–0–2	VS
			Brain injury	4,5	4–5–5–2–1–2^*^	MCS
9	Male	52	Hemorrhagic stroke	4	1–0–2–1–0–1	VS
10	Female	63	Ischemic stroke	10,5	4–5–5–2–1–2	MCS
11	Female	20	Traumatic	4	1–1–2–0–0–2	VS
			Brain injury	6	3–3–3–0–1–2^*^	MCS

Asterisks indicate the CRS-R scores relative to the second evaluation session, performed in patient 2, 7, 8, and 11.

The study was approved by the Independent Ethics Committee of the Fondazione Santa Lucia and was conducted in accordance with the Declaration of Helsinki guidelines. We obtained written informed consent from all patients' legal representatives and medical teams.

### EEG data acquisition

BAERs and auditory Event Related Potentials (ERPs) were recorded in the Post-Coma Unit at patients' bedside, as part of the routine evaluation of admitted patients. The CRS-R was administered prior to each EEG recording.

**Figure 1:**
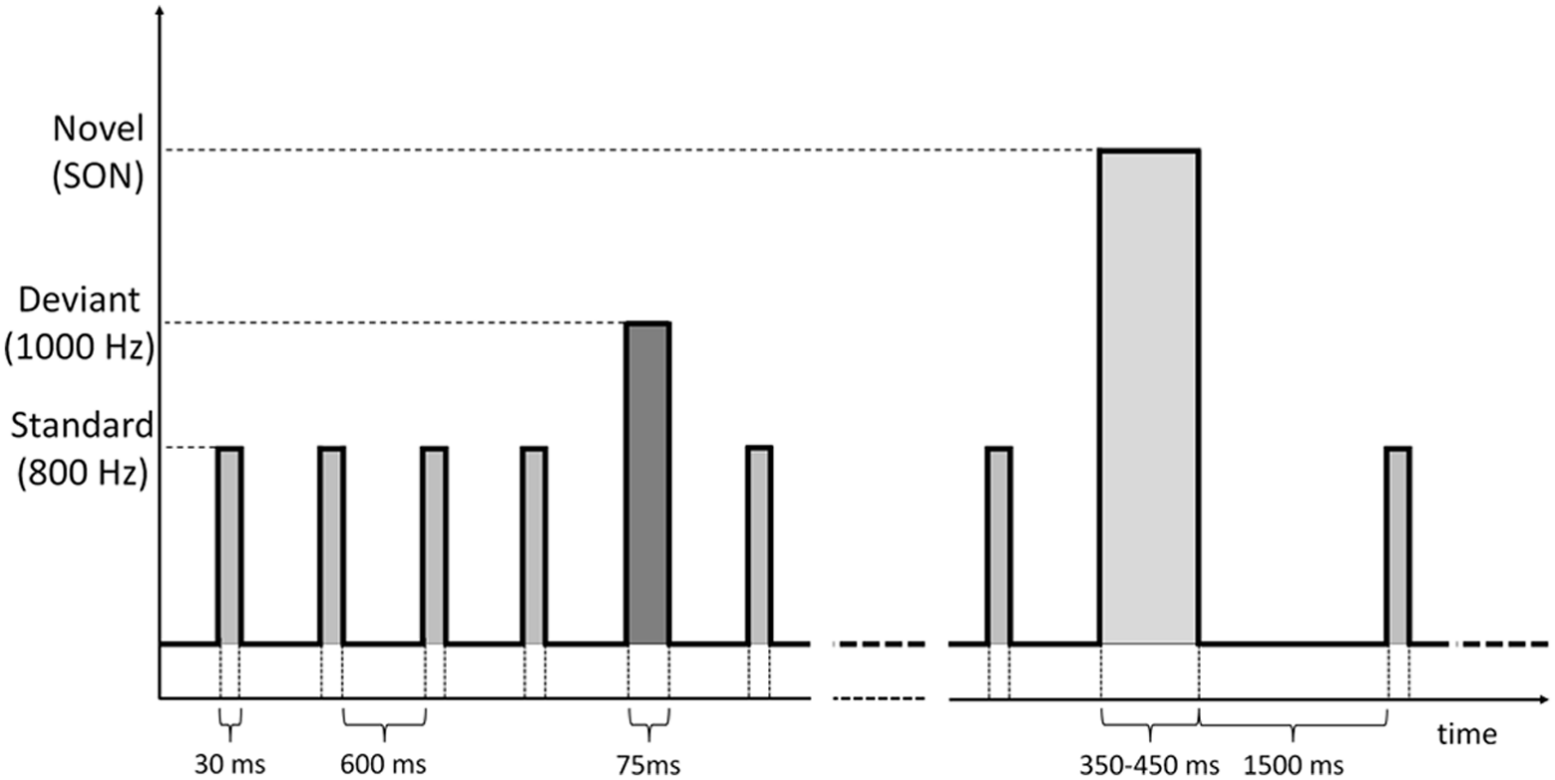
**Time sequence of standard, deviant and novel (SON) stimuli administered through the auditory oddball paradigm**.

We applied an auditory *Oddball* paradigm (Sutton et al., 1965) which included standards, deviants and the SON, digitally recorded by a female speaker using Adobe Audition software, considered as a *novel stimulus* (Fischer et al., 2008). The standard and deviant stimuli were tone bursts of 800 Hz and 1 KHz, lasting 30 ms for the standards and 75 ms for the deviants. Auditory stimuli were delivered binaurally by means of inserted earphones at an intensity of 75 dB HL. Patients were presented with 6 blocks. Each block was composed by 500 stimuli, specifically: 415 standards, 70 deviants, and 15 novel stimuli. Stimuli were pseudo-randomly presented: each deviant was preceded by 5/6 standards and between two novel stimuli there were at least 4/5 deviant stimuli. Stimulus onset asynchrony (SOA) was 600 ms for standard and deviant stimuli, whereas the standard tone burst following a novel stimulus (SON) was presented 1500 ms after the onset of the novel stimulus (duration from 350 to 450 ms). Such SOA duration was chosen in order to allow the subject to listen to the complete own name whose duration was longer than the other standard and deviant tones (Figure 1).

Stimuli sequences were programmed and delivered through the BCI2000 Software (www.bci2000.org). Two recording conditions were considered: passive where patients were asked to listen to the auditory stimuli, and active condition where they were verbally instructed to count the novel stimuli (i.e., their own name). The active blocks were always preceded by the passive, to avoid patients to persist with the counting task even when not requested.

Prior to administration of the auditory stimuli, 5 min of EEG signal were acquired (eyes-closed baseline condition) to estimate the Individual Alpha Frequency (IAF) peak, defined as the frequency associated to the strongest EEG power peak in alpha frequency range (8–13 Hz), over the posterior electrodes (Klimesch, 1999). The patients were asked to close their eyes and remain as relaxed as possible. Eyelids were maintained closed with help when necessary.

Scalp EEG potentials were continuously recorded from 10 electrode placed according to the 10–20 International System at the following positions: F3, Fz, F4, C3, Cz, C4, P3, Pz, P4, Oz (references at earlobes; impedance was kept below 5 KΩ; sampling rate was 1 KHz; Brain Amp/Vision system, Brain Products GmbH, Germany). The electro-oculogram (EOG) was recorded from two pairs of electrodes (one above and below the right eye and the other on the outer canthi of the two eyes), in order to run a semi-automatic procedure for the ocular movement artifacts removal. The patients' level of vigilance was monitored online through recording session by inspecting the EEG traces to detect signs of sleep onset.

### EEG data analysis

The EEG data were band pass filtered (1-45 Hz). Eye movement artifacts were removed from recordings based on EOG traces, by means of Gratton-Coles algorithm (Gratton et al., 1983). Other artifacts were detected with a semi-automatic procedure based on two different criteria: threshold criterion (traces which exceeded a threshold of ±80 μV were rejected) and gradient criterion (traces in which the difference between two consecutive samples exceeded ±50 μV were rejected). Data were offline filtered between 2 Hz and 20 Hz and baseline corrected in 100 ms before the stimulus onset. Responses to standard and deviant stimuli were averaged over epochs of 800 ms (including 200 ms before the stimulus onset). Responses to novel stimuli were averaged over 1700 ms duration epochs (including 200 ms before the stimulus onset). One VS patient (number 5 in Table 1) was excluded from the analysis because of the amount of artifacts due to teeth grinding (bruxism).

The ERP components identification was computed by means of a two-step process as reported in Fischer and coworkers (Guthrie and Buchwald, 1991; Fischer et al., 2008, 2010). The different ERP components were first qualitatively identified by means of the visual inspection by two neurophysiologists and successively validated by means of statistical analysis. The N100 evoked in response to standard and deviant tones was visually detected as a negative deflection (maximum peak amplitude >0.1 μV) occurring within 75–200 ms after stimulus onset. The MMN was identified as a negative deflection (maximum peak amplitude >0.75 μV) occurring within 200–350 ms after the stimulus occurrence. The novelty P300 (nP3) elicited in response to the subject's own name was visually detected as a positive deflection (maximum peak amplitude >0.75 μV) occurring between 250 and 1000 ms after the stimulus occurrence. The validation of ERP components (N100 and MMN) was performed comparing EEG epochs related to deviant and standard stimuli. Responses to deviant and novel stimuli were statistically compared to highlight the nP3 component. The waveforms were resampled by means of a bootstrap approach (Efron, 1979). The significance level of 5% was corrected by means of False Discovery Rate (FDR) to take into account multiple comparisons (Benjamini and Yekutieli, 2001). The FDR represents the expected proportion of erroneous rejections among all rejections. Considering *V* as the number of false positives (erroneous rejections) and *S* as the number of true positives (correct rejections), the FDR is given by:

(1) $$FDR=E\text{​}\left[\frac{V}{V+S}\right]$$

Let *H*_1_, *H*_2_, …, *H*_*m*_ be the null hypotheses, with *m* the number of univariate tests to be performed, and *p*_1_, *p*_2_, …, *p*_*m*_ their corresponding *p*-values. These values were ordered in increasing order (*p*_1_ ≤ *p*_2_ ≤ … ≤ *p*_*m*_) and the value *k* was chosen as the largest *i* for which:

(2) $$p_{i}\leq \frac{i}{m}\alpha$$

Therefore, the hypotheses *H*(*i*) with *i* = 1,…, *k* must be rejected and thus *p*_*i*_ represents the new adjusted significance level.

Finally, a paired *t*-test between the resampled standard and deviant (or deviant and novel for nP3) distributions was performed for each sample within the selected time window. N100 and MMN waveforms were identified when 80% of samples within the time window showed a significant difference between standard and deviant stimuli. P300 waveforms were identified when at least 62 consecutive samples (60 ms) located around the maximum (positive) amplitude peak showed a significant difference between deviant and novel stimuli within the interval of 250–1000 ms (Fischer et al., 2008).

EEG activity recorded during baseline condition was subjected to a power spectral analysis. A Fast Fourier Transformation with a 1 s Hanning window was performed on EEG data to estimate the IAF parameter defined as the frequency associated to the highest power peak in alpha frequency range over the posterior electrodes (Klimesch, 1999).

### Statistical analysis

Mean latency and amplitude values of each ERP components (N100 passive condition, MMN passive condition, nP3 passive/active conditions) at the three midline electrodes (Fz, Cz, Pz) were calculated (according to the above describe procedures): data at Cz electrode position and the mean IAF peak values were considered for the between-group analysis. The group analysis comprised the VS (*n* = 7 patients) and MCS (*n* = 7 patients, including 3 patients initially diagnosed as MCS and 4 patients who evolved from VS to MCS) groups. The Mann-Whitney *U-test* and *Wilcoxon* signed-rank *test* were applied for the between-group (N100 passive condition, MMN passive condition, nP3 passive/active conditions, IAF peaks) and for the within-group analysis (nP3 passive/active condition), respectively.

The Spearman's rank correlation coefficient was calculated to estimate possible correlation between the nP3 mean amplitude and latency values (Cz) obtained for each condition (passive/active) and each recording session, and CRS-R total scores and auditory sub-scores obtained prior to each EEG recording. (FDR; Benjamini and Yekutieli, 2001) was applied to account for multiple correlations.

A significance threshold of 5% was set for all the statistical tests. All values are reported as mean ± Standard Deviation *(SD).*

## Results

### N100 to standard tones and MMN to deviant tones

Under passive condition, we found the N100 in all patients (*VS* = −0.8 ± 0.5 μV; MCS = −0.75 ± 0.7 μV) and it was significantly delayed in VS with respect to MCS group (*VS* = 159.3 ± 10.2 ms; MCS = 124.3 ± 36.4; Mann-Whitney *U*-test, *U*_(6)_ = 35.5, *p* = 0.028). Similarly, the MMN was observed in all patients, except patient 7 (Table 1) with no significant between group differences in latency (*VS* = 339.2 ± 155.6 ms; MCS = 268.3 ± 22.6 ms) and amplitude (*VS*= −1.38 ± 0.9 μ V; MCS = −1.27 ± 0.4 μV) mean values.

### Novel P300 to subject own name

**Figure 2:**
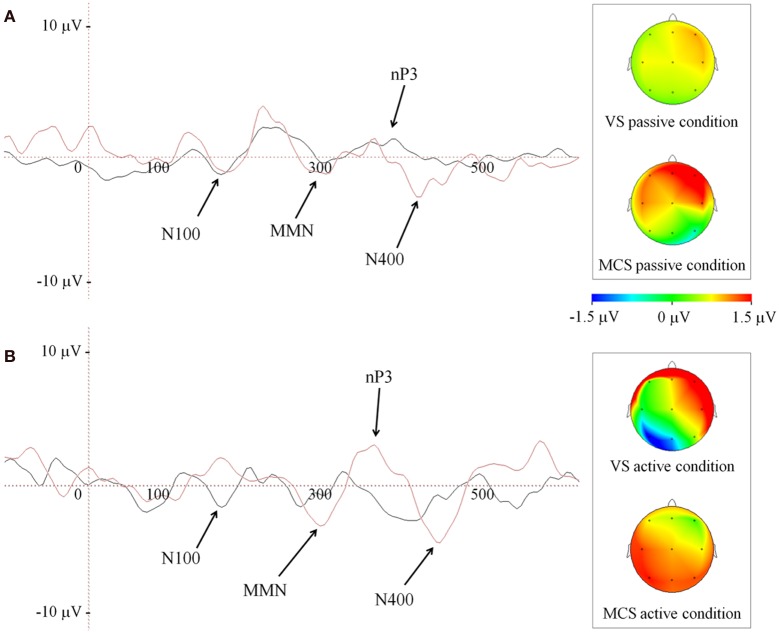
**Grand average of ERP waveforms depicted at Cz during passive (A) and active (B) listening of patients' own name (novel stimulus), in both group of patients**. Black and red traces code for VS and MCS group, respectively. The right side panels illustrate the scalp nP3 topographies at Cz. Color bar code for the amplitudes of the scalp maps obtained at the time point of ERP maximum peak.

The novel P300 (nP3) was observed in all but one patient (patient 7, in Table 2). We found a significant delay in VS with respect to MCS group, (*VS* = 515 ± 210 ms; MCS = 407.5 ± 65 ms; Mann-Whitney *U*-test, *U*_(6)_ = 26, *p* = 0.035), only under passive listening condition (Figure 2A). Active listening condition elicited an nP3 with significantly larger amplitude (Mann-Whitney *U*-test, *U*_(6)_ = 55, *p* = 0.009) in MCS (4.3 ± 2.2 μ V) as compared to VS group (1.9 ± 1 μ V; Figure 2B). Finally, we noted the presence of a clear negative deflection, occurring at 80–100 ms from the nP3 that was interpreted as an instance of the N400.

**Table 2:** **Average of the nP3 latency and amplitude values for each patient subject**.

**Patient**	**Diagnosis**	**nP3_passive**		**nP3_active**	
		**Latency (ms)**	**Amplitude (μ V)**	**Latency (ms)**	**Amplitude (μ V)**
1	MCS	345	2.2	375	3
2	VS	540	3	445	2.5
	MCS	345	3.7	300	3.4
3	VS	430	2.7	365	1.9
4	MCS	395	2.5	470	3.8
6	VS	430	2.4	415	1.1
7	VS	425	0.3	410	0.5
	MCS	455	0.9	330	0.3
8	VS	465	3.5	440	3.1
	MCS	455	5	440	6.6
9	VS	640	2	585	1.4
10	MCS	385	4.8	465	6.3
11	VS	585	1.8	585	1.8
	MCS	520	2	550	2.6

When comparing passive vs. active conditions within the two groups, statistical analysis revealed that active condition was associated with a significantly larger nP3 mean amplitude in the MCS patients (passive = 3.4 ± 1.5 μ V; active = 4.3 ± 2.2 μ V; Wilcoxon signed rank test, *W*_(6)_ = 19, *p* = 0.0425). On the contrary, the active condition elicited significantly smaller nP3 mean amplitude in the VS group (passive = 2.6 ± 1.1 μ V; active = 1.9 ± 1 μ V; Wilcoxon signed rank test, *W*_(6)_ = 17, *p* = 0.0425). No significant differences in the mean values of the nP3 latency detected in both conditions were found within patient groups.

Finally, a difference in the topography of the nP3 was observed between passive and active condition in MCS patients. As illustrated in Figure 2 (panel B, insert on the right side) the nP3 shifted from frontal toward posterior scalp regions (mainly parietal) when changing from passive to active condition.

Four patients (patient number 2, 7, 8, 11 in Table 1) underwent a second recording session timely with the change of their clinical status from VS into MCS, according to the CRS-R score. We included patient 7 even if no detectable ERP late components (MMN, nP3) were evident in the first EEG recording. The N100 component was observed in all 4 patients with no significant differences in latency (156.2 ± 12.5 ms and 112.5 ± 43.7 ms, in the first and second recording session respectively) and amplitude (−0.8 ± 0.5 and −0.9 ± 0.8 μ V, in the first and second recording session respectively). Similarly, we found the MMN component in all 4 patients (including the patient 7, with anoxic etiology) with no significant differences in latency (290 ± 52.9 and 262.5 ± 30.7 ms, in the first and second recording session respectively) and amplitude (−1.03 ± 0.2 and −1.3 ± 0.4 μ V, in the first and second recording session, respectively).

**Figure 3:**
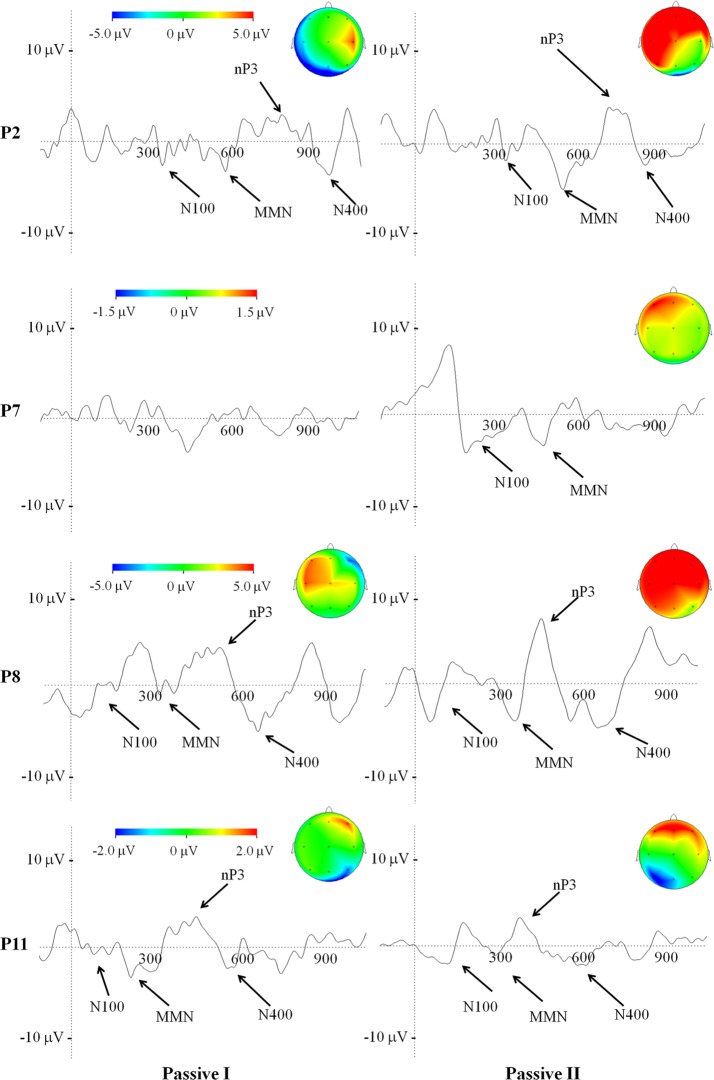
**Average of ERP waveforms and scalp potential topography depicted at Cz of passive condition related to the first and to the second recording session, in patients who underwent a change in their clinical status, according to the CRS-R**. Color bar code for the amplitudes of the scalp maps and give the size of the nP3 projections at the time point of the maximum peak.

In the second recording session as compared to the first one, we found a statistically significant increase in the nP3 amplitude, elicited by passive listening condition in patient 2 (nP3 amplitude of 3 μ V and 3.7 μ V, for the first and second recordings respectively; *p* < 0.05) and patient 8 (nP3 amplitude of 3.5 μ V and 5 μ V, for the first and second recordings, respectively; *p* < 0.05). Furthermore, the nP3 (passively) evoked during the second recording session displayed a change in the topography, being largely distributed over the frontal and posterior (parietal) scalp regions. In patient 11 and 8 no relevant changes were observed over time for both passive and active condition (Figure 3, P8 and P11).

### Power spectra analysis

The mean IAF peak value estimated at Pz was 8.1 ± 0.89 Hz and 9.8 ± 1.57 Hz for the VS and MCS group, respectively. This IAF difference was statistically significant (*Wilcoxon* signed-rank *test*; *W*_(6)_ = 17, *p* = 0.047). It is worth to note that in patients 2, 8, and 11 an increase of the IAF peak occurred between the first and second recording (*P*2 = from 7.5 to 10.5 Hz; *P*8 = 10 to 11.5 Hz; *P*11 = 8.5 to 9.5 Hz).

### Clinical and neurophysiological data correlations

**Figure 4:**
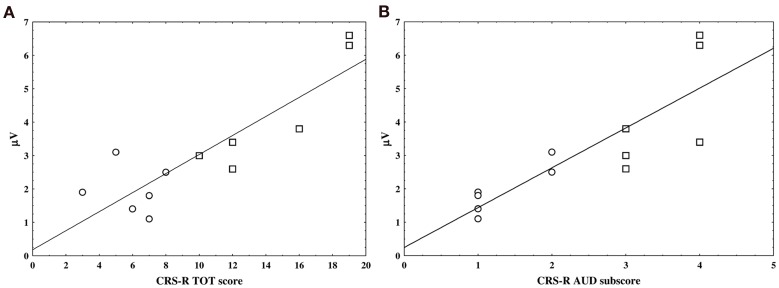
**The two scatter plots show the linear correlation between nP3 amplitude individual values in the active condition and the JFK CRS-R overall scores (panel A) and auditory sub-scores (panel B)**. VS and MCS patients are represented by circle and square, respectively.

The CRS-R total scores obtained before each EEG recording and acquired from each patient were positively correlated with the amplitude of the nP3 elicited by the SON stimuli in the active condition (*r* = 0.76, *p* = 0.004; FDR correction; Figure 4A).

Furthermore, a positive correlation was found between the auditory sub-scale scores of the CRS-R and the amplitude of the nP3 recorded under the same experimental condition (i.e., active SON) (*r* = 0.9, *p* = 0; FDR correction; Figure 4B).

## Discussion

In this longitudinal pilot study, event related potential (ERP) components elicited by an auditory stimulation paradigm administered under passive (just listening) and active (counting subject's own names, SON) condition to a sample of patients diagnosed with disorder of consciousness (DOC) were investigated and ultimately correlated with the outcome of the clinical behavioral assessment followed-up during patients' rehabilitative period. Although we are aware of the limited power of the statistical findings due to the sample size, the main findings can be summarized as follows: (i) pre-attentive ERP components, such as N100, MMN and novelty P300 (nP3), were preserved in our sample of VS and MCS patients; (ii) when patients were asked to actively count novel stimuli (i.e., SON) the nP3 component displayed a significant increase in amplitude and a wider spatial distribution with respect to the passive listening only in MCS and not in VS patients, thus suggesting a preservation of less automatic attention ability in MCS; (iii) the same nP3 component elicited by passive listening of novel stimuli in some VS patients, could show a significant amplitude increment consistently with their moving from a VS to MCS; (iv) the occipito-parietal power alpha rhythm was higher in MCS with respect to VS; (v) finally, a positive correlation between amplitude of the nP3 elicited in the active condition and total score and auditory sub-scores of the CRS-R obtained from all patients corroborates the validity of such ERP component in supporting the patient behavioral assessment that in turn, would improve the rehabilitation of such patients.

The presence of early ERP components, such as N100 indicates that the auditory sensorial register (Liegeois-Chauvel et al., 1994) was preserved in our sample of DOC patients. In line with previous evidence Glass et al., 1998; Guérit et al., 1999, the initial discrimination of the auditory target stimuli resulted to be delayed in our sample of VS as compared with MCS patients. It was suggested that prolongation of N100 latency might be related to a frontal lobe dysfunction (Jiang et al., 2000), with the prefrontal cortex being responsible for attentional resource modulation upon the sensory processing reflected by N100 (Coull, 1998). In our sample of patients, the DOC was the consequence of severe acquired brain injuries. The presence of brain lesions might have accounted for the N100 latency delay observed in VS patients, the majority of whom were at an earlier stage of the post-injury with respect to MCS.

The MMN component of ERPs did not show significant differences in mean latencies and amplitudes between the two groups of patients included in this study, thus suggesting that the automatic survey of the stimulus change (Näätänen, 1992; Näätänen et al., 2007) is rather preserved in VS and MCS patients. A similar lack of difference was reported in other studies dealing with VS and MCS patients with traumatic and non-traumatic etiology, in sub-acute and chronic stage (Kotchoubey et al., 2005; Fischer et al., 2010). The general conclusion was that such pre-attentive potential might not be selective to discriminate different DOCs. Our findings are in line with these conclusions. It is important to note that the one patient initially diagnosed as VS due to anoxia (Patient 7 in Table 1) showed a MMN component when she moved after 3 months to a MCS as indicated by the CRS-R scores. The occurrence of MMN has been reported as reliable predictor of recovery (Qin et al., 2008), particularly in the post-anoxic coma patients (Vanhaudenhuyse et al., 2008), although this etiology has been frequently reported as associated with a lack of evoked brain responses to auditory stimulation (Vanhaudenhuyse et al., 2008; Fischer et al., 2010).

The presence of a novelty P3 (nP3) elicited by SON paradigm has been already reported in patients with DOCs providing a useful tool to assess some residual cognitive functions in these behaviorally non- or minimally responsive conditions (Kotchoubey et al., 2004; Holeckova et al., 2006; Perrin et al., 2006; Fischer et al., 2008). Furthermore, in line with previous studies, (Kotchoubey et al., 2005; Perrin et al., 2006; Fischer et al., 2010), we found that all but one patient (Patient 7, Table 1) showed the nP3 component in response to a passive SON listening, regardless the clinical status (VS or MCS). As part of the P300 waveform complex, the nP3 is assumed to reflect aspects of the attentional orientation related to the working memory updating processes (Coles et al., 1988; Friedman et al., 2001), raising the question of a residual maintenance of some higher-order cognitive processing in DOC patients, yet irrespective of the clinical diagnosis. The between group comparison revealed a significant delay of such component in the VS as compared to MCS sample of patients. The observed abnormality in the nP3 latency elicited in VS with respect to MCS might be indicative of a better ability to *unconsciously* detect novel events in MCS, although the different etiology, non-homogeneity of brain lesion extension and site in our patients could account for this abnormality as well.

Similar to already existing evidence (Schnakers et al., 2008, 2009b), the mean amplitude of the nP3 in response to SON was significantly larger in MCS patients when they were asked to actively pay attention to the *novel* stimuli by counting his or her own name with respect to the solely listening to them. In the present study, we also investigated the topography of the nP3 elicited during active condition (vs. passive) and found a greater involvement of the posterior/parietal scalp areas in the MCS patients (Figure 2). Noticeably, a similar change in the nP3 topography elicited under passive condition occurred in 2 out of 4 patients (patient 2 and 8 in Table 1) who moved from VS to MCS (Figure 3). Our EEG topographical findings are concordant with previous studies that have shown the presence in MCS and VS of long-latency ERP components such as P300 family components which involve fronto-temporo-parietal cortices and backward connection between these areas, being these latter connectivity patterns considered as reliable markers for neuronal conscious perception (Del Cul et al., 2007; Garrido et al., 2007; Fischer et al., 2010; Boly et al., 2011). In this regard, it is worth to note that in our sample of MCS patients, an instance of N400 occurred in response to novel stimuli, particularly under active condition. This result can favor the idea of a preserved complex, albeit partially automatic (Perrin and Garcia-Larrea, 2003), of linguistic processing (Duncan et al., 2009) in such clinical status. Similarly, Perrin et al. (2006) reported that salient stimuli like SON could also elicit a N400 component in some DOC patients. The presence of this endogenous linguistic ERP, has been also associated with a good prognosis in consciousness disorders (Steppacher et al., 2013). Bearing in mind the caution when interpreting the presence of brain evoked responses in DOCs, the overall present findings on auditory nP3 generated under active condition, further extend previous reports in favor of a likely preserved ability in MCS patients to *consciously* orient their attention toward stimuli, following the instructions by the experimenter.

Specifically to the nature of the SON paradigm, it can be argued that the obtained responses to such stimuli are not name-specific. In fact, concomitant factors such as the lowest occurrence of the stimulus as novel, the longer stimulus duration, the complexity of its acoustic and semantic salience and the ecological salience to the subject, all contribute to the response elicited by SON. The difference in brain responses could be attributed to the meaning of the SON stimulus only if other factors were held constant, but this is not the case. The interaction of these four factors hinders the interpretation of the response to the SON and is the main limitation of the study. Anyway, even if one has to take into account the debatable name-specific nature of the SON stimulus responses, an active listening condition requiring the execution of a command (i.e., to count stimuli) still retains the value as a probe to test the patient's “following command ability” in absence of a recognizable motor output. These letters are instead, mandatory for the CRS-R clinical assessment based on execution upon verbal instructions of “simple” motor actions (like to turn the gaze, to take, to touch or to move something of his/her own body or in the environment).

On the contrary, with respect to what we have seen in MCS patients, we observed a significant decrease in the amplitude of the nP3 component evoked by SON under active as compared to passive condition, in our VS patients. We can only speculate that since the active was always preceded by the passive condition (to avoid patients to persevere in the task of counting) such opposite trend with respect to what observed in the MCS group, might be the result of fatigue and/or of limited attentional resources available in VS patients. In fact it cannot be ruled out that the overall length of the stimulation blocks (a relatively high number of trials were acquired in order to cope with the presence of artifacts) might have had a detrimental effect on the nP3 amplitude evoked in VS.

Finally, in a recent retrospective study on a cohort of 50 DOC patients, Babiloni and colleagues (Babiloni et al., 2009) have shown that the cortical alpha rhythms as measured also by means of the individual alpha peak (IAF; Klimesch, 1999) are altered in these patients according to their level of recovery from VS. In our small sample of patients, we only calculated the IAF (no source analysis was performed) and found that such EEG parameter was *normal* in both VS and MCS group of patients with a significant difference in favor of MCS (higher mean value). Even if no more advanced EEG processing is available in our study, we can consider this finding as a further surrogate of a preserved neurophysiological substrate of *conscious perception* in our sample of patients.

To our knowledge this is the first study which correlated the nP300 parameters with the CRS-R scores and reported a positive correlation between electrophysiological and clinical parameters as extracted from patients' evaluation over time. Accordingly, the amplitude of the nP3 components elicited by novel stimuli (SON) under active condition in our sample of DOC patients, positively correlated with the patient's CRS-R overall scores, being lower in VS and higher in MCS patients. As yet, such positive correlation was present between the auditory CRS-R sub-scores and the nP3 amplitude elicited in all patients during the active condition.

Although this latter finding might not be surprising since both behavioral and electrophysiological measures of the patient's ability to allocate attentional resources have exploited the auditory modality, it is of relevance that behavioral evolution of patients parallels changes in some ERP component as elicited during *voluntary* attention shifts. The correlation with the total scores of the clinical scale and the nP3 suggests that this ERP component is, however, affected by the overall level of brain functions assessed by means of the CRS-R and that thus its amplitude appears to be sensitive to the proportion of the overall improvement of single patient abilities.

The issue of correlations between clinical assessment based on behavioral tests and neuroimaging and electrophysiological data in DOC patients still remains a matter of debate. In fact, some studies did not find correlation between clinical and instrumental data (Di et al., 2007; Estraneo et al., 2013). On the other hand, more recent studies on DOC patients have found correlation between functional and structural brain connectivity patterns (at rest) and different levels of consciousness impairment as evaluated with CRS-R (Vanhaudenhuyse et al., 2010; Fernandez-Espejo et al., 2012). One plausible explanation for this still inconclusive results might be found in the non- homogeneity of patient cohorts in terms of DOC etiology (especially cerebral anoxia), time from event and site/side of brain lesions, among the different studies. Another source of discrepancy between correlation findings might be that correlations are reported with only CRS-R total scores and this has to be taken with caution as the diagnosis of DOC is based on the scores of the sub-scales and not on the overall score of the CRS-R.

Bearing in mind the limits of a pilot study, our overall findings confirm the added value of paraclinical testing based on EEG, as a means to disclose spared brain functions in DOC. Future investigations should address a follow-up longitudinal measurements in a large cohort of patients (especially the VS) to validate the predictive value of ERPs in following-up DOC evolution and to determine whether and to what extent clinical factors (i.e., the etiology, the site/size of brain lesions) can affect the several EEG parameter characteristics and their relevance in supporting the bedside management of such critical clinical conditions.

### Conflict of interest statement

The authors declare that the research was conducted in the absence of any commercial or financial relationships that could be construed as a potential conflict of interest.

## References

[B1] Andrews K. Murphy L. Munday R. Littlewood C. (1996). Misdiagnosis of the vegetative state: retrospective study in a rehabilitation unit. BMJ 313, 13–16 10.1136/bmj.313.7048.13 8664760 PMC2351462

[B2] Babiloni C. Sarà M. Vecchio F. Pistoia F. Sebastiano F. Onorati P. (2009). Cortical sources of resting-state alpha rhythms are abnormal in persistent vegetative state patients. Clin. Neurophysiol. 120, 719–729 10.1016/j.clinph.2009.02.157 19299197

[B3] Benjamini Y. Yekutieli D. (2001). The control of the false discovery rate in multiple testing under dependency. Ann. Stat. 29, 1165–1188 18298808

[B4] Bernat J. L. (2006). Chronic disorders of consciousness. Lancet 367, 1181–1192 10.1016/S0140-6736(06)68508-5 16616561

[B5] Boly M. Garrido M. I. Gosseries O. Bruno M. Boveroux P. Schnakers C. (2011). Preserved feedforward but impaired top-down processes in the vegetative state. Science 332, 858–862 10.1126/science.1202043 21566197

[B6] Coles M. G. Gratton G. Donchin E. (1988). Detecting early communication: using measures of movement-related potentials to illuminate human information processing. Biol. Psychol. 26, 69–89 10.1016/0301-0511(88)90014-2 3061481

[B7] Coull J. T. (1998). Neural correlates of attention and arousal: insight from electrophysiology, functional neuroimaging and psychopharmacology. Int. J. Neurosci. 78, 145–156 9654384 10.1016/s0301-0082(98)00011-2

[B8] Cruse D. Chennu S. Chatelle C. Bekinschtein T. A. Fernández-Espejo D. Pickard J. D. (2011). Bedside detection of awareness in the vegetative state: a cohort study. Lancet 378, 2088–2094 10.1016/S0140-6736(11)61224-5 22078855

[B9] Cruse D. Chennu S. Chatelle C. Fernández-Espejo D. Bekinschtein T. A. Pickard J. D. (2012). Relationship between etiology and covert cognition in the minimally conscious state. Neurology 78, 816–822 10.1212/WNL.0b013e318249f764 22377810 PMC3304945

[B10] Del Cul A. Baillet S. Dehaene S. (2007). Brain dynamics underlying the nonlinear threshold for access to consciousness. PLoS Biol. 5:e260 10.1371/journal.pbio.0050260 17896866 PMC1988856

[B11] Di H. B. Yu S. M. Weng X. C. Laureys S. Yu D. Li J. Q. (2007). Cerebral reponse to patient's own name in the vegetative and minimally conscious state. Neurology 68, 859–899 10.1212/01.wnl.0000258544.79024.d0 17372124

[B12] Donchin E. Ritter W. McCallum W. C. (1978). Cognitive psychology: the endogenous components of the ERP, in Event-Related Brain Potentials in Man, eds Callaway E. Tueting P. Koslow S. H. (New York, NY: Academic Press), 349–412

[B13] Duncan C. C. Barry R. J. Connolly J. F. Fischer C. Michie P. T. Näätänen R. (2009). Event-related potentials in clinical research: guidelines for eliciting, recording, and quantifying mismatch negativity, P300, and N400. Clin. Neurophysiol. 120, 1883–1908 10.1016/j.clinph.2009.07.045 19796989

[B14] Efron B. (1979). Bootstrap methods: another look at the jackknife. Ann. Stat. 7, 1–26 10.1214/aos/117634455217187989

[B15] Estraneo A. Moretta P. Loreto V. Lanzillo B. Cozzolino A. Saltalamacchia A. (2013). Predictors of recovery of responsiveness in prolonged anoxic vegetative state. Neurology 80, 464–470 10.1212/WNL.0b013e31827f0f31 23303855

[B16] Fernandez-Espejo D. Soddu A. Cruse D. Palacios E. M. Junque C. Vanhaudenhuyse A. (2012). A role for the default network in the bases of disorder of consciousness. Ann. Neurol. 72, 335–343 10.1002/ana.23635 23034909

[B17] Fischer C. Dailler F. Morlet D. (2008). Novelty P3 elicited by the subject's own name in comatose patients. Clin. Neurophysiol. 119, 2224–2230 10.1016/j.clinph.2008.03.035 18760663

[B18] Fischer C. Luaute J. Morlet D. (2010). Event-related potentials (MMN and Novelty P3) in permanent vegetative or minimally conscious states. Clin. Neurophysiol. 121, 1032–1042 10.1016/j.clinph.2010.02.005 20202899

[B19] Friedman D. Cycowicz Y. M. Gaeta H. (2001). The novelty P3: an event-related brain potential (ERP) sign of the brain's evaluation of novelty. Neurosci. Biobehav. Rev. 25, 355–373 10.1016/S0149-7634(01)00019-7 11445140

[B20] Garrido M. I. Kilner J. M. Kiebel S. J. Friston K. J. (2007). Evoked brain responses are generated by feedback loops. Proc. Natl. Acad. Sci. U.S.A. 104, 20961–20966 10.1073/pnas.0706274105 18087046 PMC2409249

[B21] Glass I. Sazbon L. Groswasser Z. (1998). Mapping cognitive event-related potentials in prolonged postcoma unawareness state. Clin. Electroencephalogr. 29, 19–30 9472422 10.1177/155005949802900109

[B22] Goldfine A. M. Schiff N. D. (2011). Consciousness: its neurobiology and the major classes of impairment. Neurol. Clin. 29, 723–737 10.1016/j.ncl.2011.08.001 22032656 PMC3222861

[B23] Goldfine A. M. Victor J. D. Conte M. M. Bardin J. C. Schiff N. D. (2011). Determination of awareness in patients with severe brain injury using EEG power spectral analysis. Clin. Neurophysiol. 122, 2157–2168 10.1016/j.clinph.2011.03.022 21514214 PMC3162107

[B24] Goldfine A. M. Victor J. D. Conte M. M. Bardin J. C. Schiff N. D. (2012). Bedside detection of awareness in the vegetative state. Lancet 379, 1701–1702 10.1016/S0140-6736(12)60714-4 22559892

[B25] Gratton G. Coles M. G. Donchin E. (1983). A new method for off-line removal of ocular artifact. Electroencephalogr. Clin. Neurophysiol. 55, 468–484 10.1016/0013-4694(83)90135-9 6187540

[B26] Guérit J. M. Verougstraete D. de Tourtchaninoff M. Debatisse D. Witdoeckt C. (1999). ERPs obtained with the auditory oddball paradigm in coma and altered states of consciousness: clinical relationships, prognostic value, and origin of components. Clin. Neurophysiol. 110, 1260–1269 10.1016/S1388-2457(99)00061-9 10423191

[B27] Guthrie D. Buchwald J. S. (1991). Significance testing of difference potentials. Psychophysiology 28, 240–244 10.1111/j.1469-8986.1991.tb00417.x 1946890

[B28] Holeckova I. Fischer C. Giard M. H. Delpuech C. Morlet D. (2006). Brain responses to subject's own name uttered by a familiar voice. Brain Res. 1082, 142–152 10.1016/j.brainres.2006.01.089 16703673

[B29] Jennett B. Teasdale G. Braakman R. Minderhoud J. Knill-Jones R. (1976). Predicting outcome in individual patients after severe head injury. Lancet 1, 1031–1034 10.1016/S0140-6736(76)92215-7 57446

[B30] Jiang C. Kaseda Y. Kumagai R. Nakano Y. Nakamura S. (2000). Habituation of event-related potentials in patients with parkinson's disease. Physiol. Behav. 68, 741–747 10.1016/S0031-9384(99)00244-9 10764905

[B31] Kalmar K. Giacino J. T. (2005). The JFK coma recovery scale-revised. Neuropsychol. Rehabil. 15, 454–460 10.1080/09602010443000425 16350986

[B32] Klimesch W. (1999). EEG alpha and theta oscillations reflect cognitive and memory performance: a review and analysis. Brain Res. Brain Res. Rev. 29, 169–195 10.1016/S0165-0173(98)00056-3 10209231

[B33] Kotchoubey B. Lang S. Herb E. Maurer P. Birbaumer N. (2004). Reliability of brain responses to the own name in healthy subjects and patients with brain damage. in Brainwaves and Mind: Recent Advances, eds Moore N. C. Arikan M. K. (New York, NY: Kjellberg, Inc), 75–80

[B34] Kotchoubey B. Lang S. Mezger G. Schmalohr D. Semmler A. Bostanov V. (2005). Information processing in severe disorder of consciousness: vegetative state and minimally conscious state. Clin. Neurophysiol. 116, 2441–2453 10.1016/j.clinph.2005.03.028 16002333

[B35] Laureys S. Schiff N. D. (2012). Coma and consciousness: paradigms (re)framed by neuroimaging. Neuroimage 61, 478–491 10.1016/j.neuroimage.2011.12.041 22227888

[B36] Lehembre R. Gosseries O. Lugo Z. Jedidi Z. Chatelle C. Sadzot B. (2012). Electrophysiological investigations of brain function in coma, vegetative and minimally conscious patients. Arch. Ital. Biol. 150, 122–139 10.4449/aib.v150i2.1374 23165873

[B37] Liegeois-Chauvel C. Musolino A. Badier J. M. Marquis P. Chauvel P. (1994). Evoked potentials recorded from the auditory cortex in man: evaluation and topography of the middle latency components. Electroencephalogr. Clin. Neurophysiol. 92, 204–214 10.1016/0168-5597(94)90064-7 7514990

[B38] Lombardi F. Gatta G. Sacco S. Muratori A. Carolei A. (2007). The italian version of the coma recovery scale-revised (CRS-R). Funct. Neurol. 22, 47–61 17509244

[B39] Monti M. M. Vanhaudenhuyse A. Coleman M. R. Boly M. Pickard J. D. Tshibanda L. (2010). Willful modulation of brain activity in disorders of consciousness. N. Engl. J. Med. 362, 579–589 10.1056/NEJMoa0905370 20130250

[B40] Näätänen R. (1992). Attention and Brain Function. Hillsdale, NJ: Erlbaum

[B41] Näätänen R. Gaillard A. W. Mäntysalo S. (1978). Early selective-attention effect on evoked potential reinterpreted. Acta Psychol. 42, 313–329 10.1016/0001-6918(78)90006-9 685709

[B42] Näätänen R. Paavilainen P. Rinne T. Alho K. (2007). The mismatch negativity (MMN) in basic research of central auditory processing: a review. Clin. Neurophysiol. 118, 2544–2590 10.1016/j.clinph.2007.04.026 17931964

[B43] Owen A. M. Coleman M. R. (2008). Detecting awareness in the vegetative state. Ann. N.Y. Acad. Sci. 1129, 130–138 10.1196/annals.1417.018 18591475

[B44] Owen A. M. Coleman M. R. Boly M. Davis M. H. Laureys S. Pickard J. D. (2006). Detecting awareness in the vegetative state. Science 313, 1402 10.1126/science.1130197 16959998

[B45] Perrin F. Garcia-Larrea L. (2003). Modulation of the N400 potential during auditory phonological/semantic interaction. Brain Res. Cogn. Brain Res. 17, 36–47 10.1016/S0926-6410(03)00078-8 12763190

[B46] Perrin F. Schnakers C. Schabus M. Degueldre C. Goldman S. Brédart S. (2006). Brain response to one's own name in vegetative state, minimally conscious state, and locked-in syndrome. Arch. Neurol. 63, 562–569 10.1001/archneur.63.4.562 16606770

[B47] Qin P. Di H. Yan X. Yu D. Laureys S. Weng X. (2008). Mismatch negativity to patient's own name in chronic disorder of consciousness. Neurosci. Lett. 448, 24–28 10.1016/j.neulet.2008.10.029 18938213

[B48a] Schnakers C. Vanhaudenhuyse A. Giacino J. Ventura M. Boly M. Majerus S. (2009a). Diagnostic accuracy of the vegetative and minimally conscious state: clinical consensus versus standardized neurobehavioral assessment. BMC Neurology 21, 9–35 10.1186/1471-2377-9-35 19622138 PMC2718857

[B48] Schnakers C. Perrin F. Schabus M. Hustinx R. Majerus S. Moonen G. (2009b). Detecting consciousness in a total locked-in syndrome: an active event-related paradigm. Neurocase 15, 271–277 10.1080/13554790902724904 19241281

[B49] Schnakers C. Perrin F. Schabus M. Majerus S. Ledoux D. Damas P. (2008). Voluntary brain processing in disorder of consciousness. Neurology 71, 1614–1620 10.1212/01.wnl.0000334754.15330.69 19001251

[B50] Squires K. C. Squires N. K. Hillyard S. A. (1975). Decision-related cortical potentials during an auditory signal detection task with cued observation intervals. J. Exp. Psychol. Hum. Percept. Perform. 1, 268–279 10.1037/0096-1523.1.3.268 1202150

[B51] Steppacher I. Eickhoff S. Jordanov T. Kaps M. Witzke W. Kissler J. (2013). N400 predicts recovery from disorder of consciousness. Ann. Neurol. 73, 594–602 10.1002/ana.23835 23443907

[B52] Sutton S. Braren M. Zubin J. John E. R. (1965). Evoked-potential correlates of stimulus uncertainty. Science 150, 1187–1188 10.1126/science.150.3700.1187 5852977

[B53] Teasdale G. Jennett B. (1974). Assessment of coma and impaired consciousness. a practical scale. Lancet 2, 81–84 10.1016/S0140-6736(74)91639-0 4136544

[B54] Tiitinen H. May P. Reinikainen K. Näätänen R. (1994). Attentive novelty detection in humans is governed by pre-attentive sensory memory. Nature 372, 90–92 10.1038/372090a0 7969425

[B55] Vanhaudenhuyse A. Laureys S. Perrin F. (2008). Cognitive event-related potentials in comatose and post-comatose states. Neurocrit. Care 8, 262–270 10.1007/s12028-007-9016-0 17990124

[B56] Vanhaudenhuyse A. Noirhomme Q. Tshibanda L. J. Bruno M. A. Boveroux P. Schnakers C. (2010). Default network connectivity reflects the level of consciousness in non-communicative brain-damaged patients. Brain, 133, 161–171 10.1093/brain/awp313 20034928 PMC2801329

[B57] Wolpaw J. R. Birbaumer N. McFarland D. J. Pfurtscheller G. Vaughan T. M. (2002). Brain-computer interfaces for communication and control. Clin. Neurophysiol. 113, 767–791 10.1016/S1388-2457(02)00057-3 12048038

